# Data on comparative studies of lineaments extraction from ASTER DEM, SRTM, and Cartosat for Jilledubanderu River basin, Anantapur district, A.P, India by using remote sensing and GIS

**DOI:** 10.1016/j.dib.2018.09.023

**Published:** 2018-09-11

**Authors:** M. Rajasekhar, G. Sudarsana Raju, R. Siddi Raju, M. Ramachandra, B. Pradeep Kumar

**Affiliations:** Department of Geology, Yogi Vemana University, Kadapa, Andhra Pradesh, India

**Keywords:** DEM Digital Elevation Model, SRTM Shuttle Radar Topography Mission, ASTER Advanced Spaceborne Thermal Emission and Reflection Radiometer, ASTER-DEM, SRTM, Cartosat DEM, Lineaments, Rose diagram, Extraction

## Abstract

The data deals with the functions that automatically extracted lineaments from the Cartosat, ASTER and SRTM of Digital Elevation Model (DEM) of different spatial resolutions, in the software ArcGIS 10.4. The extracted lineaments result shows the ASTER (Advanced Spaceborne Thermal Emission and Reflection Radiometer) DEM gives the lowest number of lineaments reflects Cartosat and SRTM (Shuttle Radar Topography Mission) DEM shows a medium number of lineaments. Cartosat DEM is most appropriate for extraction of contours precisely rather than ASTER and SRTM. This study reveals the Cartosat DEM data is best to use extraction of lineaments in the Indian provinces, offers at most comprehensive geological structural info amongst all the data sets. The extracted lineaments lengths and densities are determined by the statistical method. Based on the data generated lineament density and rose diagram. Cartosat DEM data are the best suited for studying very small areas as through geological and structural information can be mined by using this data.

**Specifications table**

**Table:** 

*Subject area*	*Lineaments structures and Remote Sensing & GIS*
*More specific subject area*	*Remote Sensing and GIS and Satellite data*
*Type of data*	*Table, figures*
*How the data were acquired*	*Toposheets from Survey of India, ASTER and SRTM DEM data from USGS website, Cartosat DEM data from NRSC Bhuvan and Field surveys*
*Data format*	*Processed and Analyzed*
*Experimental factors*	*Toposheets are georeferenced and digitized lineaments from DEM data by using ArcGIS 10.4 & ERADAS imagine the software.*
*Experimental features*	*Cartosat DEM data best for Lineaments study and SRTM data is good compared with ASTER DEM data*
*Data source location*	*77° 48′ 34′′ to 78° 58′ 11′′ E and 14° 05′ 35′′ to 14° 26′ 45′′ N*
*Data accessibility*	*The data are accessible within the article.*

**Value of the data**

• The data can serve as baseline for the lineaments structures study of the area.
• Data presented here can be used to implement ground water recharge and management.
• Data are georeferenced and digitized it can be utilized in future studies.
• It is also useful to researchers, stakeholders and hydrogeologists for aquifer management.
• The data can be useful for the socio-economic of the study area.

## 1 Data

### 1.1 Study area

Jilledubanderu basin situated in Anantapur district has been selected for the present study. The hierarchy of the river system related to the study area is shown below.

Jilledubanderu - A tributary of Maddileru

Maddileru - A tributary of Chitravathi River

Chitravathi - A tributary of river Pennar

**Fig. 1:**
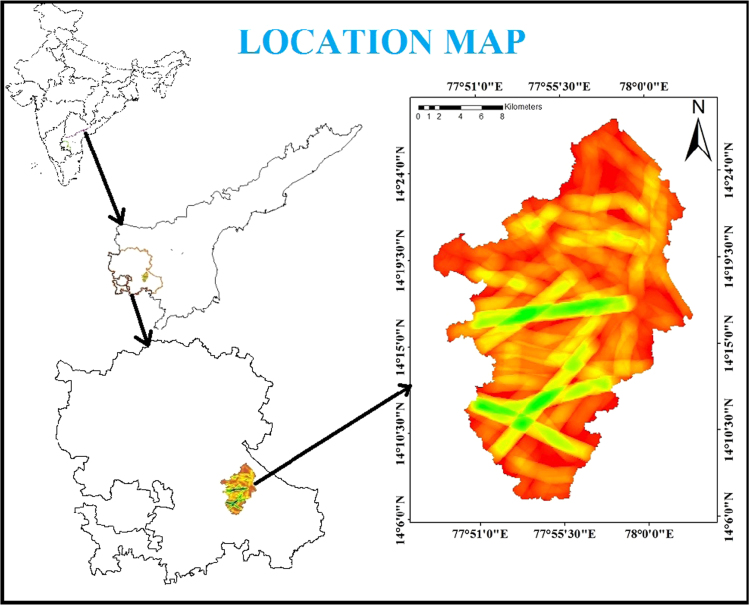
Location map of the study area with reference to Jilledubanderu River basin.

**Fig. 2:**
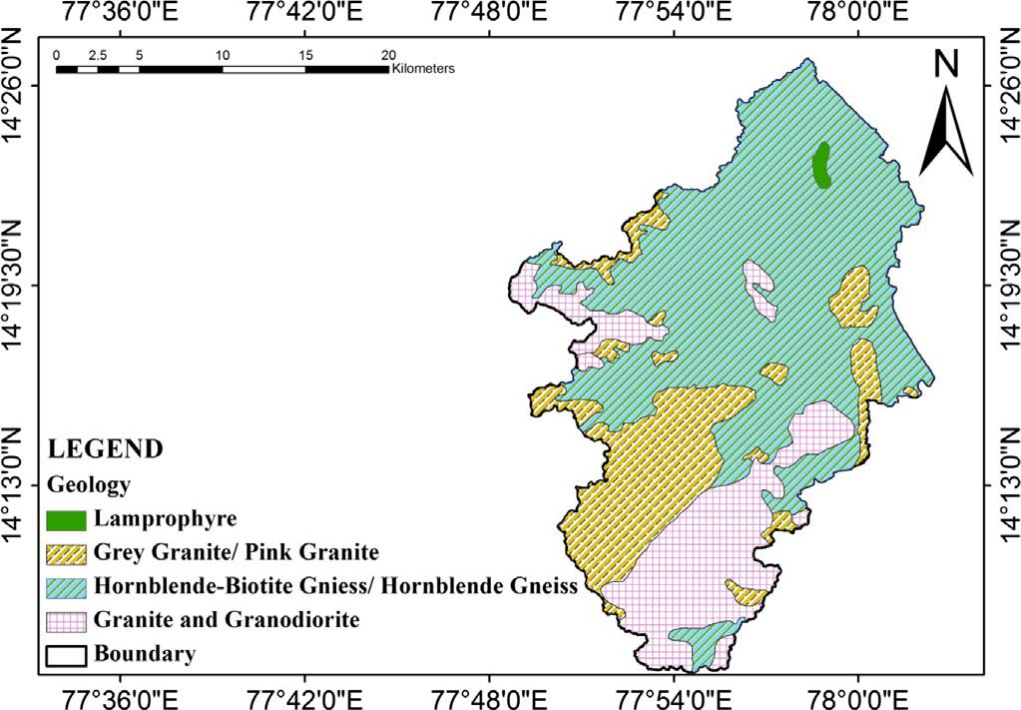
Geological map of the study area.

Jilledubanderu rises in the southeastern part of Anantapur district, a highly drought affected region. The basin is located between 77° 48′ 34′′ E longitude to 78° 58′ 11′′ E longitude and 14° 05′ 35′′ North latitude to 14° 26′ 45′′ North latitude. The entire geographical area of the river basin is 487 sqkm^2^ falling in the topo sheets of 57F/15, 57F/16, 57J/3, and 57J/4 of scale 1:50,000 and covers the Bukkapatnam, Chennekottapalle, Dharmavaram, Kothacheruvu, Mudigubba, Nallamada, and Puttaparthi Mandals in Anantapur district, Andhra Pradesh, India and corresponding schematic diagram is shown in Fig. 1. The watershed comprises a total geographical area of 486.95 sqkm and covers parts of Bukkapatnam Mandal occupies more than half of the watershed area (52%) followed by Mudigubba (27%). The study area covered Archaean Peninsular Gneissic Complex of hornblende biotite gneiss, granodiorite, and Lamprophyre (GSI, 2002). These granitic rocks are traversed by means of dolerite dykes. In the northern a part of the watershed an isolated patch of lamprophyre is a gift and corresponding geology schematic diagram is shown in Fig. 2.

## 2 Experimental design, materials, and methods

### 2.1 Data collection

The comparative studies of lineaments are studied through various datasets are ASTAER-DEM, SRTM, and Cartosat DEM in the present study. ASTER and SRTM DEM have a different spatial resolutions of 1′′ (30 m) and 3′′ (90 m). The Cartosat l DEM is developed by ISRO for indian territories having a spatial resolution of 30 m. These DEM images are geometrically ortho-corrected for higher precision of surface elevation [1]. Table 1 represents a through information and source to download these datasets.

**Table 1:** General information of data sets used in the present study.

DEM characteristics	SRTM	ASTER_DEM	CartoDEM
Tile size	50 × 50	10 × 10	
Spatial resolution	3′′ (90 m)	1′′ (30 m)	1′′ (30 m)
DEM format Geo-Tiff	WGS 84, Geo- Tiff	Geo-Tiff, WGS 84/EGM 96	Geo-Tiff, WGS 84
Coverage	600N–600S	830N–830S	830N–830S
Site to download	www.cgiar.csi.org	https://reverb.echo.nasa.gov/reverb/	http://bhuvan.nrsc.gov.in/

### 2.2 Processing of data

Now a days, geospatial technologies plays important role in the extraction of geological and structural features. Here, SRTM, ASTER, and Cartosat DEM datasets are used inthe research of shaded relief maps and geological and structural linear surface structures such as a cliff, scarp, straight valleys, straight river channels, dykes and lineaments etc. were digitized manually at 1:100,000 scale after the preparation of shaded relief maps [2], [3]. The scale was maintained at different scales can provide different terrain maps which can further lead to the differences and errors while outlining the lineaments from diverse datasets. After the removal of man-made structural features, length and frequency of all the extracted lineaments were calculated in ArcGIS 10.4 software in order to compare the data sets [4], [5], [6], [7]. Lineament density maps was prepared through ArcGIS 10.4 environment using ‘‘Line density’’ tool with the formerly extracted lineament data comparisons and analysis of lineaments was done in Rockware software for the preparation of rose diagrams [8].

### 2.3 Lineaments

**Fig. 3:**
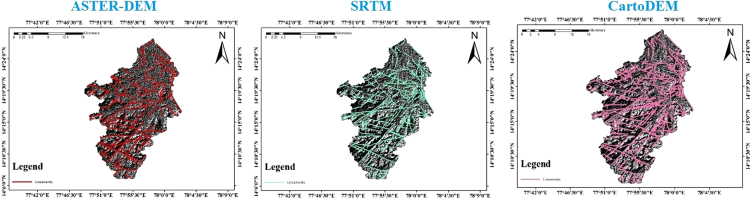
Lineaments extracted by using SRTM, ASTER, and Cartosat DEM for the study area.

**Fig. 4:**
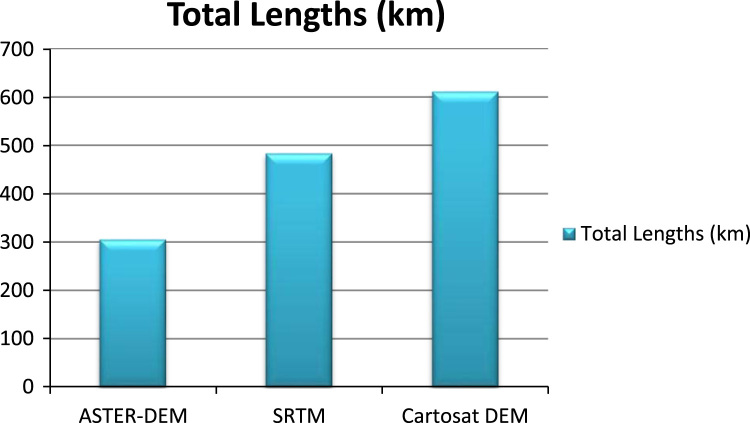
Bar-graph is showing a comparison of the total length of lineaments extracted by different datasets of the study area.

The lineaments maps are prepared from shaded relief maps through ArcGIS 10.4 software resulted from different DEMS (Fig. 3) [8], [9], [10]. Basin wise assessment of all lineament variables extracted by using SRTM, ASTER and Cartosat DEM is shown in Table 2. Observation indicates that total length of lineaments was comparatively higher inthe study area when it is drawn out by using Cartosat DEM shown in Fig. 4. Table 3 denotesentire length and frequency of lineaments for the entire study area [11], [12], [13]. Maximum number of lineaments (n = 264) are extracted from Cartosat DEM. Whereas the lowest number of lineaments could be found by using ASTER (n = 113). Therefore, it obviously appearances that more lineaments could be extracted by means of Cartosat and the least number of lineament can be drawn by means of ASTER DEM in spite of poor resolution of SRTM (n = 182), it is capable of providing a higher number of lineaments compared to the ASTER DEM [8]. These studies also reported that SRTM is better than ASTER DEM concerning surface feature extraction, such as drainage and slope [14], [15], [16], [17].

**Table 2:** Quantitative parameters of Lineaments extracted by using different data sets for the Jilledubanderu River basin.

Parameters	Jilledubanderu River basin		
	ASTER DEM	SRTM DEM	Carto DEM
Total length (km)	305.81	483.83	611.86
Maximum length (km)	14.31	15.17	15.17
Minimum Length (km)	0.18	0.18	0.18
Mean length (km)	2.71	2.66	2.32
Standard Deviation (☞)	2.58	2.46	2.23
Number of lineaments (n)	113	182	264

**Table 3:** Total length and number of lineaments extracted by different data sets for entire study area.

Parameters	ASTER-DEM	SRTM	Carto DEM
Total length (km)	305.81	483.83	611.86
Number of lineaments (n)	113	182	264

### 2.4 Lineament density

**Fig. 5:**
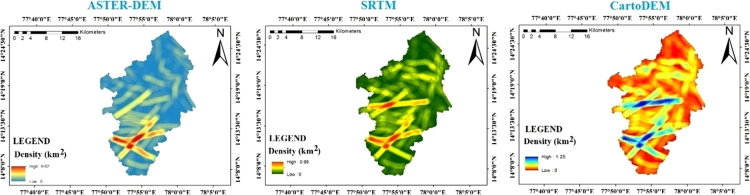
The density of the lineament fabrics extracted by using SRTM, ASTER, and Cartosat DEM for the study area.

**Fig. 6:**
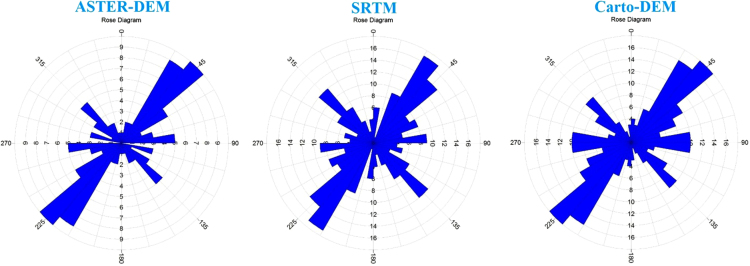
Rose diagrams represent frequency and direction of lineaments extracted by all data sets for the study area.

Lineament density can be proposed as the entire length of lineaments per unit extent.It affords a many valued geological facts about the high strength of tectonic distortion [18], rock fracturing and shearing [19], groundwater possibilities [20]. Thus, the control of lineament density is an significant and useful technique to capture many applied geological aspects and suitable datasets and careful observation offer more precision in case of lineament concentration of an area. The lineament density map of all lineaments extracted from all the data sets was related to capture the lineament concentration pattern inside the basins (Fig. 5). It is remarkably observed that for all the basins, a higher density of lineaments established in the case of Cartosat data while SRTM and ASTER data show lower density. Rose diagram has been built to associate and examines lineament directions and frequency for the study area [21] (Fig. 6). Rose diagrams mined from different data sets for a specific basin show alike trend of frequency and directions. It shows that altering of datasets does not have any considerable effect on the orientation of lineaments. In study area, orientations are mainly in N–E, S–W, NNE–SSW and NNW–SSE directions. Very limited lineaments are found to have a trend of S–E, N–W AND SSE–NNW directions.
